# Effect of the Period From COVID-19 Symptom Onset to Confirmation on Disease Duration: Quantitative Analysis of Publicly Available Patient Data

**DOI:** 10.2196/29576

**Published:** 2021-09-01

**Authors:** Myung-Bae Park, Eun Young Park, Tae Sic Lee, Jinhee Lee

**Affiliations:** 1 Department of Gerontology Division of Health and Welfare Pai Chai University Seo-gu Republic of Korea; 2 Department of Obstetrics and Gynecology Yonsei University Wonju College of Medicine Wonju Republic of Korea; 3 Department of Biomedical Science and Engineering Gwangju Institute of Science and Technology Gwangju Republic of Korea; 4 Department of Psychiatry Yonsei University Wonju College of Medicine Wonju Republic of Korea

**Keywords:** COVID-19, SARS-CoV-2, symptoms onset, duration of prevalence, confirmation, South Korea, data crawling, social media, Internet, dataset, symptom, duration, outcome, diagnosis, prevalence

## Abstract

**Background:**

In general, early intervention in disease based on early diagnosis is considered to be very important for improving health outcomes. However, there is still insufficient evidence regarding how medical care that is based on the early diagnosis of confirmed cases can affect the outcome of COVID-19 treatment.

**Objective:**

We aimed to investigate the effect of the duration from the onset of clinical symptoms to confirmation of COVID-19 on the duration from the onset of symptoms to the resolution of COVID-19 (release from quarantine).

**Methods:**

For preliminary data collection, we performed data crawling to extract data from social networks, blogs, and official websites operated by local governments. We collected data from the 4002 confirmed cases in 33 cities reported up to May 31, 2020, for whom sex and age information could be verified. Subsequently, 2494 patients with unclear symptom onset dates and 1349 patients who had not been released or had no data about their release dates were excluded. Thus, 159 patients were finally included in this study. To investigate whether rapid confirmation reduces the prevalence period, we divided the duration from symptom onset to confirmation into quartiles of ≤1, ≤3, ≤6, and ≥7 days, respectively. We investigated the duration from symptom onset to release and that from confirmation to release according to these quartiles. Furthermore, we performed multiple regression analysis to investigate the effects of rapid confirmation after symptom onset on the treatment period, duration of prevalence, and duration until release from isolation.

**Results:**

We performed multiple regression analysis to investigate the association between rapid confirmation after symptom onset and the total prevalence period (faster release from isolation). The time from symptom onset to confirmation showed a negative association with the time from confirmation to release (*t*_1_*=−*3.58; *P*<.001) and a positive association with the time from symptom onset to release (*t*_1_=5.86; *P*<.001); these associations were statistically significant.

**Conclusions:**

The duration from COVID-19 symptom onset to confirmation date is an important variable for predicting disease prevalence, and these results support the hypothesis that a short duration of symptom onset to confirmation can reduce the time from symptom onset to release.

## Introduction

### The COVID-19 Outbreak

COVID-19 was first reported on December 31, 2019, in cases of pneumonia with an unknown etiology in Wuhan, China [1]; the disease subsequently spread to neighboring countries, including South Korea and Japan [2]. On March 11, 2020, the World Health Organization (WHO) reported a total of 118,000 confirmed cases and approximately 4000 deaths due to COVID-19 in 114 countries. On the afternoon of the same day, the WHO formally declared COVID-19 to be a global pandemic [3]. The first confirmed case in South Korea, a Chinese woman in her 30s, was reported on January 20, 2020, followed by another confirmed case, a 55-year-old man who had visited Wuhan, China. Subsequently, COVID-19 appeared to be stably controlled for almost a month; however, the number of cases increased explosively after February 20, whereby South Korea became the second country after China to experience the beginning of an epidemic. Subsequently, on February 29, the daily incidence of confirmed cases peaked at 900 and has since been following a downward trend. As of November 30, 2020, a total of 64 million cumulative confirmed cases of COVID-19 had been reported worldwide [4].

### Treatment and Management of Confirmed COVID-19 Cases in South Korea

The WHO defines a confirmed case of COVID-19 as a person with a laboratory-confirmed COVID-19 infection, regardless of clinical signs and symptoms [5]. The symptoms mainly include fever, cough, shortness of breath, and breathing difficulties [6], although more than 15% of cases are asymptomatic [7]. In South Korea, individuals who have entered the country or have been in close contact with a confirmed COVID-19 case must undergo a test for COVID-19. The rapid antigen test is an efficient tool for rapid confirmation of SARS-CoV-2 infection, although it is not used as an official tool for confirmatory diagnosis in view of the capacity of laboratories to conduct the requisite number of tests and considering the number of confirmed and suspected cases. In the early stages of the COVID-19 epidemic, confirmatory testing involved two stages: pan-coronavirus and base sequencing analysis. However, the real-time polymerase chain reaction (RT-PCR) has subsequently been used for basic confirmation [8]. Individuals who are suspected of having COVID-19 based on the clinical presentation of symptoms or close contact with a confirmed patient with COVID-19 are diagnosed based on test results from two clinical samples, one each from the upper and lower respiratory tract [8,9]. RT-PCR requires at least 24 hours for the results to become available, and everyone who is tested is quarantined until the final diagnosis is determined.

The treatment strategies for COVID-19 can generally be divided into supportive care, respiratory support, symptomatic treatment, nutritional support, and psychological intervention [10]. Moreover, antibiotics can be administered to prevent secondary infections [11]. Furthermore, remdesivir is prescribed as an antiviral treatment for SARS-CoV-2, although its effectiveness is debated [12,13]. In South Korea, patients are classified according to disease severity, and patients with high severity are treated in hospitals dedicated to infectious diseases and at national inpatient treatment centers. Patients who are classified as having mild symptoms or those who do not require inpatient treatment due to improvement in clinical symptoms may be admitted to residential treatment centers. In these facilities, the medical staff monitors patients at least twice per day.

COVID-19 is highly contagious, therefore, confirmed patients must avoid contact with other people and should immediately be placed in quarantine [14-16]. In general, early intervention based on early diagnosis of the disease is considered to be very important for improving health outcomes [17-19]. However, most of the studies on COVID-19 conducted to date have focused on preventing the spread of the disease by quickly identifying confirmed patients and minimizing their contact with other people [15,20]. In contrast, there is very little evidence of how medical care that is based on the early diagnosis of confirmed cases of COVID-19 can affect the treatment outcomes. Accordingly, we aimed to investigate the effects of the duration from the onset of clinical symptoms to confirmation on the duration from the onset of clinical symptoms to the resolution of COVID-19 (release from quarantine).

## Methods

### Data Collection

**Figure 1:**
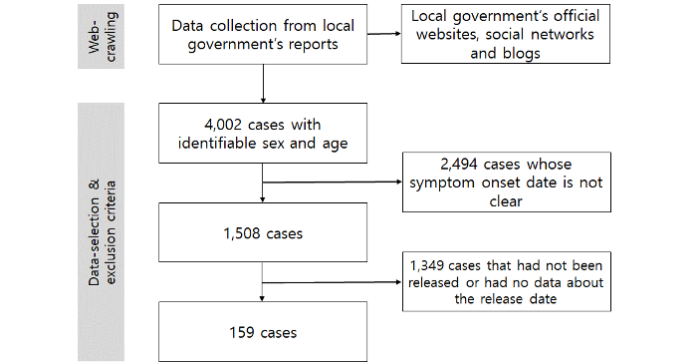
Flowchart of the data extraction process.

South Korea comprises 228 si (cities), gun (counties), and gu (districts), with 17 metropolitan city and province levels, including Seoul, its capital. All local governments disclose basic information on official websites about confirmed cases. Almost all Korean local governments have provided anonymized public information regarding confirmed COVID-19 cases, such as symptom date, release date, and age, up to the first half of 2020. We performed web crawling using Python’s Selenium module and additionally used the Beautiful Soup and Pandas libraries to collect data. In the early stage of the pandemic, the local government deidentified the information of the confirmed persons with COVID-19 and disclosed their movements, region, and age. At this time, many people connected to the local government homepages to check these data, and the server was overloaded. Accordingly, many local governments provided information to their official social networks and blogs to distribute data traffic and improve access to information. Therefore, we only collected information on local government official websites, social networks, and blogs, and we did not use information from other sites that are not guaranteed to be reliable. For preliminary data collection, we performed data crawling to extract data from social networks, blogs, and official websites operated by local governments. Second, the data were manually reviewed and revised. Data obtained in this manner underwent a final review and revision process that included the use of various check codes to complete the final data set. Data were extracted and reviewed from June 1-7, 2020 (Figure 1).

### Study Design and Participants

We collected data from the 4002 confirmed cases from 33 cities reported up to May 31, 2020, for whom sex and age information could be verified. Subsequently, 2494 patients with an unclear symptom onset date and 1349 patients who had not been released or had no data about their release date were excluded. Thus, 159 patients were finally included in this study.

A confirmed case of COVID-19 was defined based on a confirmed diagnosis from positive RT-PCR results on 2 or more clinical specimens, regardless of clinical signs and symptoms [21]. For a patient to be released from isolation, at least 7 days must pass from the day of confirmation; the patient must be afebrile without the administration of any antipyretic agents; and the clinical symptoms must show an improving trend. In addition, the patient must test negative on 2 consecutive PCR tests, each conducted at least 24 hours apart [22].

### Statistical Analysis

We performed descriptive analysis of the mean, minimum, and maximum values for the durations from symptom onset to release, symptom onset to confirmation, and confirmation to release according to the sex and age of the patients. To investigate whether rapid confirmation reduces the prevalence period, we divided the period from symptom onset to confirmation into quartiles, 1Q, 2Q, 3Q, and 4Q, of ≤1, ≤3, ≤6, and ≥7 days, respectively. We investigated the durations from symptom onset to release and confirmation to release according to these quartiles. Furthermore, we performed multiple regression analysis to investigate the effects of rapid confirmation after symptom onset on the treatment period, duration of prevalence, and duration until release from isolation.

To measure the predictive power of duration from symptom onset to release, we used logistic regression (LR) after setting the duration from symptom onset to release and other variables (age, sex, and symptom onset to confirmation) as the dependent and independent variables, respectively. We transformed the number of days from symptoms to release into tertiles (T1, T2, and T3). Two scenarios were evaluated: (1) the classification of early symptom onset to release after setting T1 and T2-T3 as 1 and 0 coding, respectively, and (2) the classification of late symptom onset to release after setting T3 and T1-T2 as 1 and 0 coding, respectively. We randomly divided the data into training and test sets by ratios of 70% and 30%, respectively. We determined two lists of variables with the following combinations: (1) age + sex and (2) age + sex + duration from symptom onset to confirmation. We constructed an LR model using a training set to predict early or late symptom onset to release. Thereafter, we measured the predictive performance of early or late symptom onset to release in the test sets. With 100 iterations of the random division of training and test sets, we measured the average performance of the classification for early or late symptom onset to release. For the 2-tailed *t* tests, we considered a *P* value <.05 to be statistically significant.

### Ethical Approval

This study was conducted in accordance with the Declaration of Helsinki, and all of the materials used in the article were publicly available data. Moreover, all of those data are nonidentifying data, and they are available for use by anyone.

## Results

The study population (N=159) included 67 men (42.1%) and 92 women (57.9%), whereas the age groups appeared in the following order: 40-59 years (n=71, 44.7%), 20-39 years (n=59, 37.1%), ≥60 years (n=21, 13.2%), and 0-19 years (n=8, 5.0%). The mean duration from symptom onset to confirmation was 6.1 days, and the mean duration from confirmation to release was 25.3 days. The mean duration from symptom onset to confirmation was shorter among females (5.6 days) than among males (6.7 days), whereas the mean duration from confirmation to release was similar for males (25.4 days) and females (25.2 days). The mean duration from symptom onset to release was 32.1 and 30.8 days among male and female patients, respectively. With regard to age, the mean duration from symptom onset to confirmation was longest in the 0-19 years group (7.9 days), followed by the 40-59 years (7.3 days), 20-39 years (5.0 days), and ≥60 years (4.3 days) groups. The mean duration from confirmation to release was longest in the ≥60 years group (27.8 days), whereas the mean duration from symptom onset to release was longest in the 40-59 years group (33 days; Table 1).

**Figure 2:**
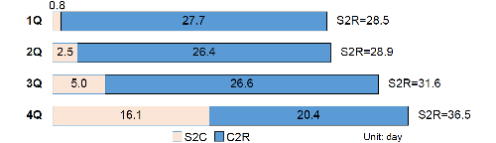
The duration from the confirmation date to release (C2R) and from the symptom onset to release (S2R) according to the quartile of the duration from symptom onset to confirmation (S2C) quartile. 1Q: ~1 day (mean age: 42.5 years), 2Q: 2–3 days (mean age: 43.8 years), 3Q: 4-6 days (mean age: 39.5 years), and 4Q: ≥7 days (mean age: 42.6 years). 1Q: first quartile; 2Q: second quartile; 3Q: third quartile; 4Q; fourth quartile; C2R: duration from confirmation date to release; S2C: duration from symptom onset to confirmation; S2R: duration from symptom onset to release.

Both the duration from confirmation to release and the duration from symptom onset to release were investigated by quartiles of duration from symptom onset to confirmation, and 4Q, 3Q, 2Q, and 1Q were ≥7 days, 4-6 days, 2-3 days, and 1 day, respectively. The duration of symptom onset to release was shortest, at 28.5 days, when the duration of symptom onset to confirmation was 1Q (≤1 day); this was followed sequentially by 2Q, 3Q, and 4Q, with 28.9, 31.6, and 36.5 days, respectively. The results indicated that the prevalence increased with increasing duration of symptom onset to confirmation (Figure 2).

**Table 1:** Characteristics of confirmed COVID-19 cases (N=159) and their durations from symptom onset to confirmation, confirmation to release, and symptom onset to release.

Characteristic		Value, n (%)	Dependent variable			Independent variables				
			Duration of symptom onset to confirmation			Duration of confirmation to release			Duration of symptom onset to release	
			Mean (SD)	Range	Mean (SD)		Range	Mean (SD)		Range
Total sample		N/A^a^	6.1 (8.9)	−1 to 48	25.3 (12.0)		4 to 72	31.4 (12.8)		7 to 73
**Sex**										
	Male	67 (42.1)	6.7 (10.4)	−1 to 48	25.4 (12.7)		7 to 72	32.1 (13.4)		11 to 73
	Female	92 (57.9)	5.6 (7.6)	0 to 47	25.2 (11.6)		4 to 69	30.8 (12.4)		7 to 70
**Age (years)^b^**										
	0-19	8 (5.0)	7.9 (11.0)	−1 to 33	22.3 (12.9)		12 to 44	30.1 (15.7)		14 to 54
	20-39	59 (37.1)	5.0 (6.6)	0 to 48	24.4 (12.1)		4 to 69	29.4 (13.3)		7 to 70
	40-59	71 (44.7)	7.3 (11.0)	0 to 47	25.7 (11.7)		7 to 72	33 (11.9)		14 to 73
	≥60	21 (13.2)	4.3 (3.6)	0 to 12	27.8 (12.9)		6 to 54	32.1 (13.6)		9 to 60

^a^N/A: not applicable.

^b^Mean 42 years (SD 14.7), range 1 to 81.

We performed multiple regression analysis to investigate the association between rapid confirmation after symptom onset and the total prevalence period (faster release from isolation). Sex and age did not show a significant association with the duration from confirmation to release or the duration from symptom onset to release. However, the duration from symptom onset to confirmation showed a negative association with the duration from confirmation to release (*t*_1_=−3.58; *P*<.001) and a positive association with the duration from symptom onset to release (*t*_1_=5.86; *P*<.001); these associations were statistically significant (Table 2).

**Figure 3:**
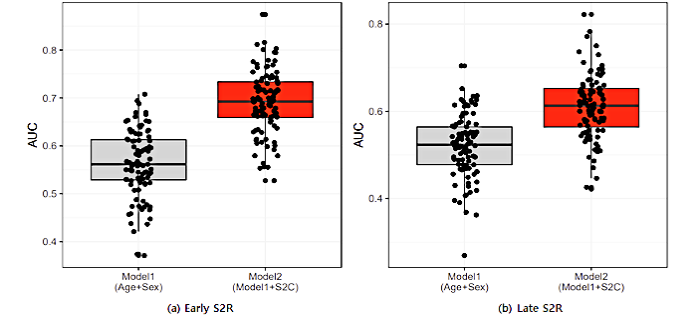
Predictive power determined through logistic regression after setting S2C and other variables (age, sex, and S2C). The analysis comprises 100 iterations by the random division of training and test data sets. AUC: area under the curve; S2C; duration from symptom onset to confirmation; S2R: duration from symptom onset to release.

We measured the informative power of the duration from symptom onset to confirmation to predict early or late symptom onset to release. We found that the duration from symptom onset to confirmation resulted in improved performance for both models (*P*<.001 for both the early and late symptom onset to release models) compared to the model that used age and sex as input features (Figure 3).

**Table 2:** Association between the durations from symptom onset to confirmation, confirmation to release, and symptom onset to release based on the results of multiple regression analysis.

Characteristics and categories		Duration from confirmation to release^a^		Duration from symptom onset to release^b^	
		T *(df)*	*P* value	T *(df)*	*P* value
**Sex**					
	Male (reference) versus female	−0.55 (1)	.58	−0.55 (1)	.58
**Age (years)**					
	≥60 (reference) versus 40-59	0.30 (1)	.76	0.30 (1)	.76
	≥60 versus 20-39	0.83 (1)	.41	0.83 (1)	.41
	≥60 versus 0-19	0.94 (1)	.34	0.94 (1)	.34
Duration of symptom onset to confirmation (days)		−3.58 (1)	<.001	5.86 (1)	<.001
*F*		2.99	.01	7.66	<.001

^a^*R*^2^=0.089; adjusted *R*^2^=0.059.

^b^*R*^2^=0.200; adjusted *R*^2^=0.174.

## Discussion

### Principal Findings

Rapid diagnosis of confirmed cases enables prompt infection control and environmental decontamination of patients with suspected infection and places, such as environmental decontamination of related places and the management of close contacts [9]. Most of the studies published to date have tended to approach the early detection of COVID-19 from the perspective of prevention of the infection and spread of the disease [23]. Reducing the time to isolation through rapid diagnosis has been proven to help prevent the spread of SARS-CoV-2 infection [15,21,23]. In this context, studies published to date have claimed that early detection of COVID-19 is important because it can lower the mortality rate [24,25]. However, this claim is an inference based on general medical knowledge about pneumonia and complications and is not rooted in actual COVID-19 data.

Through linear regression analysis, we verified that the duration from symptom onset to confirmation is a factor that influences the duration from symptom onset to release. In addition, we used LR to test the predictors of duration from symptom onset to release, and we found that the inclusion of the duration from symptom onset to confirmation as a variable in the model significantly increased the predictive power. Therefore, the duration from symptom onset to confirmation is an important variable for predicting prevalence, and these results support the hypothesis that a short duration from symptom onset to confirmation can reduce the duration from symptom onset to release. Consequently, this study demonstrated that rapid diagnosis leads to faster release from isolation. If COVID-19 could be diagnosed early, the symptoms and progression of the disease could be controlled with only simple treatment, and the reduced number of patients with severe disease could result in stable availability of hospital beds [20]. In the current COVID-19 pandemic, confirmed COVID-19 patients in many countries are not receiving proper treatment because of a shortage of medical resources; thus, efficient use of limited medical resources is extremely important. Under such circumstances, treating patients and releasing them from isolation as quickly as possible is one of the most important strategies for infectious disease control. Clinically, shortening the treatment period could be one of the best methods to improve the personal safety and quality of life of patients as well as the safety of medical staff and to reduce the workload of medical staff.

Any person with symptoms or suspicion of SARS-CoV-2 infection should be tested immediately, and intervention must take place as soon as possible upon confirmation. Delays in the confirmation of COVID-19 increase the social burden of the spread of this infectious disease but also contribute to the clinical burden of an increase in the prevalence period. The results of the regression analysis in this study showed that sex and age were unassociated with the prevalence and isolation periods of patients with COVID-19. These findings are not consistent with a previous study which reported that the prognosis of COVID-19 is influenced by age [10]. An earlier study focused on the qualitative aspect of patients’ health or medical condition after treatment or release from isolation [10], whereas this study investigated the prevalence period. Therefore, a direct comparison is difficult because of the differences in the level of variables. The COVID-19-related mortality rate is higher among the older population [5,26]; therefore, rapid diagnosis is important for older persons. The older population has a high likelihood of having underlying diseases and onset of complications, whereas the risk of disease progression to severe conditions is also higher. Thus, higher mortality is to be expected and, therefore, early intervention through rapid confirmation is expected to reduce the mortality rate [27]. Consequently, considering the results of this and previous studies, rapid confirmation could shorten the prevalence period, lead to better prognosis, and reduce the mortality rate. Along with the advancement in the diagnosis of COVID-19, such as nucleic acid tests, among which the PCR method is considered as the “gold standard” for detection of the virus, rapid confirmation is very important to not only prevent the spread of disease but also enable better patient care.

This study has some limitations. First, we could not distinguish between severely and mildly ill patients, and it is possible that the prevalence period may differ between these two groups. Second, the study only considered a quantitative variable of duration of symptom onset to release, instead of clinical outcomes such as complications and posttreatment prognosis. Third, a sufficiently large sample size could not be obtained. Fourth, because we only used crawled data available on the web, other factors besides the duration from symptom onset to confirmation that could affect the duration from symptom onset to release could have resulted in uncorrected bias in the results of the study. Nonetheless, in this study, we obtained information on the confirmed diagnosis date for every patient, although many cases did not have a symptom onset date. This was due to the fact that many patients could not accurately remember their symptom onset date. To overcome these challenges, efforts to secure a record on the date of symptom onset in the clinical field are necessary, and additional studies should be conducted that consider disease severity and treatment.

### Conclusion

The duration from symptom onset to the date of confirmation of a disease is an important variable for predicting prevalence, and our results support the hypothesis that a short duration from symptom onset to confirmation of COVID-19 can reduce the duration from symptom onset to release. Consequently, this study demonstrated that rapid diagnosis leads to faster release from isolation.

### Data Availability

To obtain the processed data, please contact the authors to request the data.

## Abbreviations

LR: logistic regression
RT-PCR: real-time polymerase chain reaction
WHO: World Health Organization
